# Who is willing to participate in low-risk pragmatic clinical trials without consent?

**DOI:** 10.1007/s00228-017-2332-1

**Published:** 2017-09-12

**Authors:** Rafael Dal-Ré, Antonio J. Carcas, Xavier Carné

**Affiliations:** 1 Clinical Research, BUC (Biosciences UAM+CSIC) Program, International Campus of Excellence, Universidad Autónoma de Madrid, Ciudad Universitaria de Cantoblanco, Madrid, Spain; 2 Chair of Bioethics “Grifols Foundation”, University of Vic - Central University of Catalonia, Vic, Barcelona Spain; 3 Present Address: Epidemiology Unit, Health Research Institute-Fundación Jiménez Díaz University Hospital, Universidad Autónoma de Madrid, Avda. Reyes Católicos 2, E-28040 Madrid, Spain; 4 Clinical Pharmacology Department, La Paz University Hospital, IdiPaz, School of Medicine, Universidad Autónoma de Madrid, Madrid, Spain; 5 Clinical Pharmacology Department, Clínic Hospital, August Pi i Sunyer Biomedical Research Institute (IDIBAPS); Clinical Fundamentals Department, Universidad de Barcelona, Barcelona, Spain

**Keywords:** General notification, Written informed consent, Pragmatic randomized controlled trial, Survey, Learning health care system, Low-intervention clinical trial

## Abstract

**Purpose:**

General notification offers a possible alternative to written informed consent for pragmatic randomized controlled trials (pRCTs). It involves patients being informed through brochures, posters, and letters that research is being conducted simultaneously to providing clinical care and that patients will be enrolled in pRCTs without study-specific consent. A previous survey found that a substantial minority of respondents endorsed general notification. We aimed to know who is willing to enroll in this type of trials using general notification rather than written consent.

**Methods:**

The previous study was a cross-sectional, probability-based survey, with a 2 × 2 factorial design. Two scenarios were assessed: two low-risk pRCTs in hypertension, one comparing two drugs with similar benefit/risk ratio and the other taking the same drug in the morning or at night. Each scenario had two routes: written consent vs verbal consent and written consent vs general notification. In this study, we were interested in the latter route in both scenarios. Respondents’ preferences were measured based on their recommendation to the research ethics committee and the respondent’s personal preference. We aimed to investigate the characteristics of those supporting general notification in either outcome or the variables explaining consistency and inconsistency between their personal preference and their recommendation. Based on the results of the original survey, we aimed to have at least 200 inconsistent respondents; to this end, the sample size was increased accordingly in a second wave of the survey.

**Results:**

One thousand six hundre and ten respondents were included; 1003 from the original survey and 607 new ones belonging to the second wave. Thirty-nine percent of respondents chose general notification as personal preference and/or recommendation. Respondents with lower education levels were more prone to accept general notification than those holding a university degree [OR (95% CI)], primary school [2.959 (2.069–4.232)], secondary school [2.899 (2.09–4.021)], or high school [1.620 (1.184–2.217)]. Also unemployed [1.372 (1.064–1.770)] and retired [1.445 (1.049–1.990)], but not students, showed preference for general notification in comparison with those employed. Individuals more than 24 years old and having received high school or university (or postgraduate) education were statistically significantly more consistent in their decisions.

**Conclusions:**

Thirty-nine percent of respondents is open to not to be asked for their informed consent in low-risk pRCTs; of these, those being less educated and not having current job or being retired are significantly more open to general notification. The use of this alternative method to written consent for simultaneous conduct of pRCTs and care should be considered and educational programs settled up to, in the case of public acceptance, ensure its ethical appropriateness.

**Electronic supplementary material:**

The online version of this article (10.1007/s00228-017-2332-1) contains supplementary material, which is available to authorized users.

The new EU clinical trials regulation [1], that will come into force in 2019, introduces, for the first time ever, a new type of randomized controlled trial, the so-called low-intervention trial (Table 1). This type of trial is, in many instances, equivalent to pragmatic randomized controlled trial (pRCT). pRCT is one of the most useful designs to conduct comparative effectiveness research. However, seeking written informed consent may jeopardize their conduct: the too time-consuming informed consent process hinders recruitment [2]. Furthermore, understanding of different elements of information provided in the informed consent process is consistently poor, raising concerns on whether many participants’ decision-making is meaningful [3].

**Table 1:** Low-intervention clinical trial definition as per EU clinical trials regulation [1]

Low-intervention clinical trial is a clinical trial which fulfills all of the following conditions
(a) The investigational medicinal products, excluding placebos, are authorized
(b) According to the protocol of the clinical trial, (i) the investigational medicinal products are used in accordance with the terms of the marketing authorization; or (ii) the use of the investigational medicinal products is evidence-based and supported by published scientific evidence on the safety and efficacy of those investigational medicinal products in any of the Member States concerned; and
(c) The additional diagnostic or monitoring procedures do not pose more than minimal additional risk or burden to the safety of the subjects compared to normal clinical practice in any Member State concerned

A number of experts have suggested that alternatives should be considered such as verbal consent and the so-called “general notification” [4–6]. In the former, the investigator verbally informs the potential participant on the key aspects of the trial and after answering all questions, seeks his/her consent to include the individual in the trial. In the latter, no specific information on a given pRCT is provided to potential participants: patients will be informed in general through posters, letters and brochures that studies are being conducted and recruited without being asked for their informed consent for specific studies. Through a common survey, the acceptance by American and Spanish adults of these two alternatives (verbal consent and general notification) vs written informed consent has been assessed. In this survey, two scenarios (two-drug pRCT and dose-timing pRCT of the same drug; in hypertensive patients) were assessed; in both of them, respondents should decide whether they will recommend to a research ethics committee (REC) written consent or verbal consent, and written consent or general notification; they should also choose what method they would prefer for themselves. The alternative methods were chosen by 37% [7] and 23% [8] of Americans and Spaniards, respectively. Of note is that general notification was chosen by 40% of Spaniards and was more popular (2.4 times higher) than verbal consent (17%). These results prompted us to investigate the characteristics of those adults who preferred and/or would recommend general notification rather than written informed consent. In addition, since respondents had to decide about their personal preference and their recommendation to a REC, we investigated what were the variables explaining consistency and inconsistency of these two decisions.

## Participants and methods

The design and conduct of the survey have been explained elsewhere [8]. The survey was administered to individuals belonging to Netquest (GfK group) panel, Spain. This panel comprises almost 200,000 people. Potential panelists are invited to join (“single-use” invitation); any adult Spaniard with Internet access could be invited. This is a probability-based online closed panel to which potential members are accepted to join with the goal of ensuring that is representative of the non-institutionalized civilian Spanish population [with the exception of the oldest (≥ 75 years) age group]. Panelists receive non-survey-specific incentives through a point-based reward program; points can be exchanged for more than 1200 different items such as books, cosmetics, and household electrical appliances.

The survey started by explaining a hypothetical hospital in which all patients were informed through letters, brochures, and posters on the simultaneous provision of care and the conduct of research. This was a cross-sectional survey, with a 2 × 2 factorial design. Two scenarios were assessed: two low-risk pRCTs in hypertension, comparison of two drugs with similar risk/benefit ratio or taking the same drug in the morning or at night. Each scenario had two routes: written consent vs verbal consent and written consent vs general notification. Each respondent was given the survey with one of the four routes. In the original survey, each of the four routes had some 500 respondents [7]. In this study, we were interested in the two routes having the opportunity to choose between general notification and written consent, one belonging to the drug pRCT and the other to the dose-timing pRCT.

The primary outcome measures were the respondent’s recommendation to the REC (“If you were to give advice to the REC, would you recommend written consent or general notification?”) and the respondent’s preference (“If you were a patient in this hospital, which would you personally prefer, written consent or general notification?”). In some cases, responses to both questions resulted in discrepancies between recommendation to the REC and personal preference. Thus, two groups were considered: “consistent” (recommendation was identical to personal preference) and “inconsistent” (recommendation was different to personal preference).

Following the results of the original survey [8], we estimated that a sample size of 200 respondents in the inconsistent group would provide 80% power to detect an 8% absolute difference between groups, assuming a two-sided level of 0.05.

The sample distribution of sociodemographic variables and diagnostic and control for hypertension variables in the two scenarios (drug pRCT and dose-timing pRCT) was compared in order to assess representativeness of the sample in each group with that of the general population and to ensure the absence of statistically significant differences between the two scenarios.

To analyze characteristics of respondents choosing general notification, two different groups were created: those choosing general notification as personal preference and/or recommendation to the REC and those that always chose written consent. Consistency in respondents’ decisions was dichotomized in two categories: “consistent” and “inconsistent.” To assess the association between characteristics of respondents and choice of general notification/written consent and consistency/inconsistency, the Pearson chi-square test of independence corrected for bootstrap and conditional logistic regression models were used. Conditional logistic regression models were used to analyze probability of choosing general notification according to perceptions and to analyze probability of consistency according to perceptions.

All analyses were conducted in IBM SPSS statistics, version 21. According to final sample distribution, poststratification weights were not used. Statistical significance was defined as a *p* value < 0.05; all tests were two-sided.

## Results

The original survey was forwarded to 3298 panel members with 2008 respondents (response rate 60.9%); 1003 (of 1649) of these 2008 respondents had to choose between written consent and general notification in both scenarios (drug pRCT and dose-timing pRCT) [8]. To complete the sample size needed for the present study, the survey was forwarded to 1006 additional panel members with 607 respondents that were added to the 1003 initially included in the original survey belonging to the two routes of interest. We end up with 1610 respondents [response rate 60.6% (1610/2655); 802 in drug pRCT and 808 in dose-timing pRCT] and with 1372 (85%) and 238 (15%) respondents in the consistent and inconsistent groups, respectively.

Sampling distribution of sociodemographic variables did not show statistically significant differences between groups according to scenario (Supplemental information-1). A majority of respondents (61%) preferred and/or recommended to the REC written informed consent. Respondents choosing general notification as personal preference and/or recommendation to the REC (*n* = 629; 39%) got similar percentages in both scenarios (Table 2). Of these 629 respondents, 85% were consistent in their responses when deciding on their personal preference and the recommendation to the REC, whereas 15% were inconsistent, providing different responses (Table 2).

**Table 2:** Consistency between respondents’ recommendation to the research ethics committee and personal preferences

Variable	Overall, *N* (%)	Drug pRCT, *N* (%)	Dose-timing pRCT, *N* (%)
	(*n* = 1.610) [95% CI]	(*n* = 802) [95% CI]	(*n* = 808) [95% CI]
Recommended written consent, preferred written consent	981 (60.9) [58.6%; 63.6%]	507 (63.2) [59.8%; 66.9%]	474 (58.7) [54.8%; 62.0%]
Recommended general notification, preferred general notification	391 (24.3) [22.1%; 26.6%]	176 (21.9) [18.9%; 24.9%]	215 (26.6) [23.4%; 29.7%]
**Total consistent**	**1372 (85.2)** **[83.4%; 87.0%]**	**683 (85.1)** **[82.8%; 87.6%]**	**689 (85.3)** **[82.6%; 87.5%]**
Recommended written consent, preferred general notification	123 (7.6) [6.4%; 8.8%]	61 (7.6) [5.8%; 9.3%]	62 (7.7) [6.0%; 9.7%]
Recommended general notification, preferred written consent	115 (7.1) [5.9%; 8.4%]	58 (7.2) [5.5%; 9.0%]	57 (7.0) [5.5%; 8.9%]
**Total inconsistent**	**238 (14.7)** **[13.0%; 16.6%]**	**119 (14.8)** **[12.4%; 17.2%]**	**119 (14.7)** **[12.5%; 17.4%]**
No statistically significant differences by scenario (*p* = 0.172)			
General notification was the personal preference and/or recommendation	629 (39.1) [36.8%; 41.5%]	295 (36.8) [33.1%; 40.2%]	334 (41.3) [38.0%; 45.2%]
Written consent was the personal preference and the recommendation	981 (60.9) [58.6%; 63.6%]	507 (63.2) [59.8%; 66.9%]	474 (58.7) [54.8%; 62.0%]

No statistically significant differences by scenario (*p* = 0.061)

*N* no. of respondents, *95% CI* 95% confidence interval, *pRCT* pragmatic randomized controlled trial

Comparison of the characteristics of respondents choosing always written consent with those that supported general notification as personal preference and/or recommendation to REC and between consistent and inconsistent respondents (univariate analyses) showed differences (*p* < 0.10) in a number of variables: gender, age, marital status, annual household income, employment status, geographical area, and education (Supplemental information 2a and 2b, respectively).

Those variables showing *p* values < 0.10 in the univariate analyses were included in the multivariate logistic regression analyses that showed that those respondents not having university degrees and that were unemployed or retired were statistically significantly more prone to choose general notification as a personal preference and/or recommendation to the REC (Table 3). On the other hand, respondents more than 24 years old and having received high school or university (and postgraduate) education were statistically significantly more consistent in their decisions (Table 4).

**Table 3:** Logistic regression results on respondents’ sociodemographic characteristics that have chosen general notification as personal preference and/or recommendation to the research ethics committee

Variable^a^	*N*, (%)^b^	OR^c^ [95% CI]	*p* value
Age (years)			
18–24	80 (41.2)	1	
25–34	87 (31.4)	0.705 [0.433; 1.149]	0.161
35–44	133 (37.0)	0.795 [0.483; 1.309]	0.367
45–54	131 (38.9)	0.862 [0.518; 1.435]	0.568
55–64	114 (46.0)	0.958 [0.555; 1655]	0.879
> 64	84 (43.1)	0.932 [0.485; 1.792]	0.833
Education			
Primary school	154 (51.2)	2.959 [2.069; 4.232]	< 0.001
Secondary education	215 (49.4)	2.899 [2.090; 4.021]	< 0.001
High school	185 (33.6)	1.620 [1.184; 2.217]	0.003
University degree or postgraduate	76 (23.4)	1	
Employment status			
Employed	248 (32.6)	1	
Unemployed or other	224 (47.5)	1.372 [1.064 1.770]	0.015
Retired	100 (46.5)	1.445 [1.049; 1.990]	0.024
Student	57 (35.2)	1.133 [0.788; 1.630]	0.501
Marital status			
Never married	163 (38.6)	1	
Married	304 (39.0)	0.746 [0.564; 0.987]	0.040
Living with partner or other	162 (39.6)	0.854 [0.632; 1.155]	0.306
Annual household income			
No answer	137 (37.6)	1	
No income	38 (51.4)	1.696 [0.989; 2.909]	0.055
< 12.600 €	112 (50.9)	1.388 [0.967; 1.992]	0.076
12.600–25.000 €	200 (40.1)	1.096 [0.815; 1.473]	0.544
25.001–38.000 €	82 (32.5)	0.964 [0.670; 1.386]	0.844
38.001–50.000 €	39 (31.7)	1.103 [0.691; 1.759]	0.682
> 50.000 €	21 (26.9)	1.066 [0.593; 1.915]	0.831
Ideology			
No answer	117 (43.0)	1	
Left	120 (41.1)	1.060 [0.748; 1.500]	0.744
Left or moderate left	101 (31.4)	0.683 [0.482; 0.968]	0.032
Moderate	197 (40.5)	0.983 [0.719; 1.342]	0.912
Right or moderate right	61 (38.1)	0.979 [0.643; 1.490]	0.921
Right	33 (42.9)	1.050 [0.620; 1.779]	0.856
Geographical area			
North	93 (37.1)	1.103 [0.682; 1.784]	0.691
Northeast	117 (37.1)	1.107 [0.694; 1.764]	0.670
East	85 (39.9)	1.126 [0.690; 1.839]	0.635
Central west	160 (37.0)	1.062 [0.677; 1.666]	0.792
South	134 (46.7)	1.602 [1.003; 2.560]	0.049
Islands	40 (35.7)	1	

*N* no. of respondents, *95% CI* 95% confidence interval

^a^Conditional logistic regression including sex, age, employment status, education, and annual income (only variables with *p* < 0.10 in the univariate analysis were included)

^b^Have chosen general notification

^c^Adjusted odds ratio

**Table 4:** Logistic regression results on respondents’ sociodemographic characteristics among consistent respondents that have chosen general notification

Variable^a^	*N*, (%)^b^	OR^c^ [95% CI]	*p* value
Gender			
Female	641 (83.2)	1	
Male	731 (87.0)	1.291 [0.970; 1.718]	0.080
Age (years)			
18–24	148 (76.3)	1	
25–34	236 (85.2)	1.692 [1.022; 2.645]	0.03
35–44	308 (85.8)	1.991 [1.276; 3.140]	0.003
45–54	299 (88.7)	2.650 [1.683; 4.395]	< 0.001
55–64	212 (85.5)	2.244 [1.389; 3.787]	0.002
≥ 64	169 (86.7)	2.068 [1.289; 3.770]	0.009
Education			
Primary school	250 (83.1)	1	
Secondary education	354 (81.5)	0.892 [0.616; 1.346]	0.567
High school	481 (87.3)	1.557 [1.077; 2.422]	0.033
University degree or postgraduate	288 (88.6)	1.778 [1.177; 2.867]	0.017
Employment status			
Student	128 (79.0)	1	
Employed	672 (88.3)	1.026 [0.564; 1.868]	0.932
Unemployed or other	389 (82.4)	0.898 [0.483; 1.671]	0.735
Retired or disabled	183 (85.1)	0.687 [0.302; 1.561]	0.370
Marital status			
Never married	342 (81.0)	1	
Married	678 (87.0)	1.384 [0.918; 2.087]	0.121
Living with partner or other	352 (86.1)	1.395 [0.932; 2.088]	0.106
Annual household income			
No answer	307 (84.3)	1	
No income	55 (74.3)	0.642 [0.346; 1.193]	0.161
< 12.600 €	177 (80.5)	0.858 [0.546; 1.348]	0.505
12.600–25.000 €	429 (86.0)	1.154 [0.779; 1.711]	0.475
25.001–38.000 €	226 (89.7)	1.402 [0.837; 2.350]	0.199
38.001–50.000 €	108 (87.8)	1.113 [0.588; 2.106]	0.742
> 50.000 €	70 (89.7)	1.312 [0.578; 2.980]	0.517
Ideology			
No answer	227 (83.5)	1	
Left	251 (86.0)	1.126 [0.702; 1.805]	0.623
Left or moderate left	283 (87.9)	1.308 [0.812; 2.109]	0.270
Moderate	418 (85.8)	1.096 [0.721; 1.665]	0.668
Right or moderate right	133 (83.1)	0.849 [0.493; 1.462]	0.555
Right	60 (77.9)	0.605 [0.318; 1.149]	0.125

*N* no. of respondents, *95% CI* 95% confidence interval

^a^Conditional logistic regression including sex, age, employment status, education, and annual income (only variables with *p* < 0.10 in the univariate analysis were included)

^b^Were consistent

^c^Adjusted odds ratio

## Discussion

The first finding to be mentioned is that a majority of respondents (61%) recommended the REC and/or preferred to be asked for written informed consent when a hypothetical pRCT is run in hospitals where research and care are simultaneously conducted. This should be highlighted even when some elements of information (risks and side effects, placebo, and freedom to withdraw) are poorly understood and have not changed over 30 years, despite many attempts made to improve the inform consent process quality [3]. In the search for alternatives to the standard (long) written informed consent, a recent large, international, randomized trial has shown that using a concise consent form provided no benefits (with regard to participants’ comprehension of information and satisfaction) over the use of the standard consent form [9].

With regard to the specific objectives of this study, there are three main findings arising. First, a substantial minority (39%) of respondents have recommended to the REC and/or preferred not to be asked for their written informed consent in low-risk pRCTs. Since respondents were asked to place themselves in a hypothetical scenario, we cannot know what would be the actual percentage of respondents that would not ask for written informed consent in the actual health care system where no trial is conducted at the same time to providing care without asking for specific trial consent. However, although being a minority, the figure is remarkable. If it is confirmed in surveys conducted in patients and in other EU countries, National Health Services within the EU should seriously consider informing their citizens on the importance of the simultaneous conduct of pRCTs and health care. This should be followed by the assessment of general notification as an acceptable method to conduct pRCTs in different settings (hospitals and primary care). If accepted by patients, this will significantly ease the conduct of pRCTs embedded in clinical practice and will improve recruitment of trial participants, a serious hurdle in current RCTs [10, 11].

Second, it seems reasonable to argue that those individuals supporting general notification in pRCTs could be more prone to trust the hospital (i.e., the healthcare system) in which they receive medical care. The two characteristics influencing choosing general notification were to be less educated and to have no current job or being retired. These two characteristics could define a specific group as compared to those individuals having better education and being employed. In countries with public universal national healthcare system, publicly funded educational programs—through letters, brochures, posters, and even TV and radio—on the conduct of research simultaneously to the provision of health care will be critical. In the long run, if a majority of the population would be willing to accept general notification as an appropriate approach in low-risk pRCTs, regulators, RECs, and investigators should be convinced that individuals are well informed on the simultaneous provision of health care and the conduct of research to consider general notification as an ethically acceptable approach.

Finally, consistency in the responses with regard to personal preference and recommendation to the REC was associated to age and better education. This suggests that the above mentioned educational programs should have specific plans targeted to young (18–24 years old) adults—mainly those with no high school education. This age group very rarely needs to be attended in health care services and hence will have less chance to be informed through the standard educational programs.

Unfortunately, the US investigators who conducted this same survey did not analyze the characteristics associated to the Americans choosing general notification instead of written consent. In any case, our findings should be confirmed through surveys conducted in patients and check if those patients willing to support general notification have the same characteristics to those observed in our study. Limited data from the US suggest that a majority of patients support alternatives to written informed consent [12]. This should be confirmed in Spain and in other EU countries before starting to consider general notification as an ethically appropriate alternative to written informed consent for pRCTs.

## Electronic supplementary material

ESM 1 (PDF 449 kb).

ESM 2 (PDF 334 kb).

ESM 3 (XLSX 186 kb).
