# Supplementary material for: Who is willing to participate in low-risk pragmatic clinical trials without consent?
Source: Eur J Clin Pharmacol. 2017 Sep 12;73(12):1557–63. doi: 10.1007/s00228-017-2332-1 (PMC5684310; doi:10.1007/s00228-017-2332-1)
Supplement: Supplementary file 1 — (PDF 449 kb). [file 228_2017_2332_MOESM1_ESM.pdf]

Rafael Dal-Ré* (a,b), Antonio J Carcas (c), Xavier Carné (d)

(a) Clinical Research, BUC (Biosciences UAM+CSIC) Program, International Campus of Excellence, Universidad Autónoma de Madrid, Ciudad Universitaria de Cantoblanco, Madrid, Spain;
(b) Chair on Bioethics "Grifols Foundation", University of Vic - Central University of Catalonia, Vic, Barcelona, Spain.
(c) Clinical Pharmacology Department, La Paz University Hospital, IdiPaz, School of Medicine, Universidad Autónoma de Madrid, Madrid, Spain.
(d) Clinical Pharmacology Department, Clínic Hospital, August Pi i Sunyer Biomedical Research Institute (IDIBAPS); Clinical Fundamentals Department, Universidad de Barcelona; Barcelona.

*Rafael.dalre@quironsalud.es

Supplemental information-1

Characteristics of Survey Respondents

	Overall	General Notification, %	
	(n= 1610)	Drug pRCT (n=802)	Dose-Timing pRCT (n=808)
**Age**			
18/24 y	194 (12.0)	12.2	11.9
25/34 y	277 (17.2)	17.3	17.1
35/44 y	359 (22.3)	22.1	22.5
45/54 y	337 (20.9)	20.7	21.2
55/64 y	248 (15.4)	15.7	15.1
65/74 y	165 (10.2)	10.0	10.5
≥ 75 y	30 (1.9)	2.0	1.7
**Sex**			
Male	840 (52.2)	53.9	50.5
Female	770 (47.8)	46.1	49.5
**Geographical area**			

North	251 (15.6)	16.1	15.6
Northeast	315 (19.6)	19.9	19.6
East	213 (13.2)	13.4	13.2
Central-West	432 (26.8)	26.6	26.8
South	287 (17.8)	17.7	17.8
Islands	112 (7.0)	6.3	7
**Marital status**			
Never married	422 (26.2)	27.3	25.1
Married	779 (48.4)	46.9	49.9
Divorced	99 (6.1)	7.0	5.3
Separated	23 (1.4)	1.1	1.7
Widowed	24 (1.5)	1.6	1.4
Living with partner	235 (14.6)	14.1	15.1
No answer	28 (1.7)	2.0	1.5
**Annual Household income**			
< 12.600 €	220 (13.7)	14.3	13
12.600 - 25.000€	499 (31.0)	29.9	32.1
25.001 - 38,000€	252 (15.7)	15.3	16
38,001 - 50,000€	123 (7.6)	7.5	7.8
> 50.000 €	78 (4.8)	5.2	4.5
No income	74 (4.6)	4.2	5
No answer	364 (22.6)	23.4	21.8
**Employment status**			
Employed	761 (47.3)	46.4	48.1
Unemployed or other	472 (29.3)	29.3	29.3
Retired	215 (13.4)	14.1	12.6
Student	162 (10.1)	10.2	9.9

**Education**			
Primary school	301 (18.7)	17.5	19.9
Secondary education	433 (26.9)	26.9	26.9
High school	551 (34.2)	33.4	35.0
University and postgraduate	325 (20.2)	22.2	18.2
**Religious attendance**			
Regularly	180 (11.2)	11.1	11.3
Rarely	324 (20.1)	21.4	18.8
Never	949 (58.9)	57.2	60.6
No answer	157 (9.8)	10.2	9.3
**Ideology**			
1 Extreme left	47 (2.9)	2.6	3.2
2	245 (15.2)	15.3	15.1
3	322 (20.0)	20.2	19.8
4 Moderate	487 (30.2)	28.9	31.6
5	160 (9.9)	10.8	9
6	51 (3.2)	3	3.3
7 Extreme right	26 (1.6)	1.4	1.9
No answer	272 (16.9)	17.7	16.1
**Diagnosed with hypertension**			
Yes	456 (28.3)	29.2	27.5
No	1100 (68.3)	67.5	69.2
I Dont know	40 (2.5)	2.5	2.5
No answer	14 (0.9)	0.9	0.9
**Prescription treatment for hypertension**			
Yes, currently	262 (57.5)	56.0	59.0

Yes but no currently	61 (13.4)	14.1	12.6
No	132 (28.9)	29.9	27.9
No answer	1 (0.2)	0.0	0.5

pRCT: pragmatic randomized controlled trial
