# Supplementary material for: Who is willing to participate in low-risk pragmatic clinical trials without consent?
Source: Eur J Clin Pharmacol. 2017 Sep 12;73(12):1557–63. doi: 10.1007/s00228-017-2332-1 (PMC5684310; doi:10.1007/s00228-017-2332-1)
Supplement: Supplementary file 2 — (PDF 334 kb). [file 228_2017_2332_MOESM2_ESM.pdf]

Rafael Dal-Ré* (a,b), Antonio J Carcas (c), Xavier Carné (d)

(a) Clinical Research, BUC (Biosciences UAM+CSIC) Program, International Campus of Excellence, Universidad Autónoma de Madrid, Ciudad Universitaria de Cantoblanco, Madrid, Spain;
(b) Chair on Bioethics "Grifols Foundation", University of Vic - Central University of Catalonia, Vic, Barcelona, Spain.
(c) Clinical Pharmacology Department, La Paz University Hospital, IdiPaz, School of Medicine, Universidad Autónoma de Madrid, Madrid, Spain.
(d) Clinical Pharmacology Department, Clínic Hospital, August Pi i Sunyer Biomedical Research Institute (IDIBAPS); Clinical Fundamentals Department, Universidad de Barcelona; Barcelona.

*Rafael.dalre@quironsalud.es

Supplemental information-2

2a.Characteristics of respondents by option

	**General notification was the personal preference *and/or* recommendation, % (n=629)**	**Written consent was the personal preference *and* the recommendation, % (n=982)**	**p value**
**Age**			
18/24 y	12.7	11.6	**0.027**
25/34 y	13.8	19.4	
35/44 y	21.1	23.0	
45/54 y	20.8	13.7	
55/64 y	18.1	9.5	
65/74 y	11.4	8.8	
≥ 75 y	1.9	1.8	
**Sex**			
Male	51.8	52.4	0.824
Female	48.2	47.6	

**Geographical area**			
North	14.8	16.1	0.099
Northeast	18.6	20.6	
East	13.5	13.0	
Central-West	25.4	27.7	
South	21.3	15.6	
Islands	6.4	7.3	
**Marital status**			
Never married	25.9	26.4	**0.012**
Married	48.3	48.4	
Living with partner	12.2	16.1	
Other	13.5	9.1	
**Annual Household income**			
< 12.600 €	17.8	11.0	**0.000**
12.600 - 25.000€	31.8	30.5	
25.001 - 38.000€	13.0	17.3	
38.001 - 50.000€	6.2	8.6	
> 50.000 €	3.3	5.8	
No income	6.0	3.7	
No answer	21.8	23.1	
**Employment status**			
Employed	39.4	52.3	**0.000**
Unemployed or other	35.6	25.3	
Retired	15.9	11.7	
Student	9.1	10.7	
**Education**			

Primary school	24.5	15.0	**0.014**
Secondary education	34.0	22.3	
High school	29.4	37.3	
College and postgraduate	12.1	25.4	
**Religious attendance**			
Regularly	11.0	11.3	0.183
Rarely	21.8	19.1	
Never	56.1	60.8	
No answer	11.1	8.9	
**Ideology**			
1 Extreme left	2.9	3.0	0.057
2	16.2	14.6	
3	16.1	22.5	
4 Moderate	31.3	29.6	
5	9.7	10.1	
6	2.9	3.4	
7 Extreme right	2.4	1.1	
No answer	18.9	15.8	
**Diagnosed with hypertension**			
Yes	30.5	26.9	0.102
No	65.3	70.2	
I Dont know	3.3	1.9	
No answer	0.8	0.9	
**Prescription treatment for hypertension**			
Yes. currently	56.8	58.0	0.809
Yes but no currently	13.0	13.6	
No	30.2	28.0	

No answer	0.0	0.4	

2b.Characteristics of Consistent and Inconsistent Respondents

	Consistent, % (n=1372)	Inconsistent, % (n=238)	p value
**Age**			
18/24 y	10.8	19.3	**0.012**
25/34 y	17.2	17.2	
35/44 y	22.4	21.4	
45/54 y	21.8	16.0	
55/64 y	15.5	15.1	
65/74 y	10.5	8.8	
≥ 75 y	1.8	2.1	
**Sex**			
Male	53.3	45.8	**0.035**
Female	46.7	54.2	
**Geographical area**			
North	15.3	17.2	0.791
Northeast	19.4	20.6	
East	13.0	14.3	
Central-West	27.3	24.4	
South	17.8	18.1	
Islands	7.2	5.5	
**Marital status**			
Never married	24.9	33.6	**0.043**
Married	49.4	42.4	
Living with partner	14.8	13.4	
Other	10.9	10.6	

**Annual Household income**			
< 12.600 €	12.9	18.1	**0.008**
12.600 - 25.000€	31.3	29.4	
25.001 - 38.000€	16.5	10.9	
38.001 - 50.000€	7.9	6.3	
> 50.000 €	5.1	3.4	
No income	4.0	8.0	
No answer	22.4	23.9	
**Employment status**			
Employed	49.0	37.4	**0.003**
Unemployed or other	28.4	34.9	
Retired	13.3	13.4	
Student	9.3	14.3	
**Education**			
Primary school	18.2	21.4	**0.014**
Secondary education	25.7	33.6	
High school	35.1	29.4	
College and postgraduate	21.0	15.5	
**Religious attendance**			
Regularly	11.2	10.9	0.110
Rarely	19.6	23.1	
Never	60.0	52.9	
No answer	9.2	13.0	
**Ideology**			
1 Extreme left	3.1	1.7	0.076

2	15.2	15.5	
3	20.6	16.4	
4 Moderate	30.5	29.0	
5	9.7	11.3	
6	3.1	3.4	
7 Extreme right	1.2	3.8	
No answer	16.5	18.9	
**Diagnosed with hypertension**			
Yes	28.6	26.5	0.112
No	68.4	67.6	
I Dont know	2.1	4.6	
No answer	0.8	1.3	
**Prescription treatment for hypertension**			
Yes. currently	58.3	52.4	0.797
Yes but no currently	13.2	14.3	
No	28.2	33.3	
No answer	0.3	0.0	

pRCT: pragmatic randomized controlled trial
